# Frequency and Dynamics of Non-motor Symptoms Presentation in Hispanic Patients With Parkinson Disease

**DOI:** 10.3389/fneur.2019.01197

**Published:** 2019-11-14

**Authors:** Claudia Marisol Sánchez-Martínez, José Alberto Choreño-Parra, Noel Placencia-Álvarez, Lilia Nuñez-Orozco, Parménides Guadarrama-Ortiz

**Affiliations:** ^1^Department of Neurology, Centro Médico Nacional 20 de Noviembre, ISSSTE, Mexico City, Mexico; ^2^Centro Especializado en Neurocirugía y Neurociencias México (CENNM), Mexico City, Mexico; ^3^Escuela Nacional de Ciencias Biológicas, Instituto Politécnico Nacional, Mexico City, Mexico

**Keywords:** Parkinson disease, non-motor symptoms, NMSQuest, autonomic symptoms, depression

## Abstract

The clinical phenotype of Parkinson's disease (PD) encompasses a wide range of non-motor symptoms (NMS) compromising the quality of life of affected patients. Currently, information about NMS in PD is scarce among Hispanic populations. Furthermore, few studies have reported the temporal pattern of NMS presentation. We conducted a cross-sectional study aimed to describe the frequency and time of NMS occurrence in Hispanic patients with PD using the self-completed NMS questionnaire (NMSQuest). Participants were interrogated about the time of each NMS presentation respect to the onset of motor symptoms. The frequency of NMS was described according to gender, age at disease onset, disease duration and Hoehn and Yahr (H&Y) stage. We enrolled 120 patients, 73.33% males and 26.66% females, with a mean age of 63.33 ± 8.60 years. All the participants presented at least 1 NMS. The median number of NMS per patient was 12. The most frequent NMS domains were miscellaneous, urinary tract, sleep/fatigue, and gastrointestinal tract symptoms, with no significant gender differences. The most frequent individual NMS were nocturia, urinary urgency, feeling sadness, and constipation. Any patient reported NMS before the onset of motor manifestations. The pattern of occurrence of NMS domains in our population was as follows: attention/memory, cardiovascular, gastrointestinal tract, perceptual problems/hallucinations, mood/cognition, urinary, miscellaneous, sleep/fatigue, and sexual function. Nausea/vomiting was the earliest symptom observed in all patients, whereas sexual dysfunction and changes in interest for sex were the last symptoms to occur. We found no differences in the total number and frequency of NMS between participants grouped according to their age at disease onset. Conversely, patients with a duration of disease >10 years reported a higher frequency of NMS compared to participants with a duration of disease < 10 years. The total number of NMS per patient increased as the HY stage progressed. The proportion of patients presenting symptoms of the gastrointestinal tract, urinary tract, mood/cognition, cardiovascular, and sexual function domains was higher in the HY 4–5 group. Our study provides relevant data to improve our understanding of NMS in PD, which may contribute to anticipate and plan diagnostic and therapeutic strategies among Hispanic PD patients.

## Introduction

Parkinson's disease (PD) is a leading cause of neurological disability due to the progressive development of incapacitating motor symptoms in affected patients. Cardinal features of PD, such as resting tremor, bradykinesia, rigidity, and postural instability, result from the functional disruption of neuronal circuits connecting basal ganglia with motor areas of the cortex which are regulated by dopamine (1). Nonetheless, PD is also characterized by the presence of accompanying dysregulations in autonomous, sensitive, sleep, and cognitive functions that lead to the appearance of a wide spectrum of non-motor symptoms (NMS) (2).

Currently, it is widely accepted that not only motor manifestations, but also NMS exert a negative impact on the quality of life (QoL) of PD patients (3–5). However, NMS were unappreciated for many years remaining under-targeted for mechanism-specific therapeutic purposes. Conversely, several pharmacological agents enhancing dopamine production within the central nervous system exist for the management of motor PD symptoms (6). This remarks the necessity for a better understanding of the pathophysiological aspects and clinical phenotype of NMS among different populations of individuals with PD. In this regard, most of what is currently known about the frequency of NMS in PD comes from studies in white Caucasians (3–5, 7–11). However, ethnic differences in the prevalence and clinical phenotype of motor features in PD have been described in the past (12), indicating that the genetic background may also influence the occurrence of NMS. Despite this, little literature exists about the incidence of NMS in non-Caucasian groups of PD patients. Furthermore, most of the studies that have addressed the frequency of NMS have not described the dynamics of NMS presentation during the course of PD. Thus, a better knowledge of the temporal pattern of NMS presentation would allow physicians to anticipate their occurrence and to plan their management, with the ultimate goal of limiting their detrimental effect on the QoL of patients with PD.

In the current study we evaluated the frequency and temporal pattern of NMS presentation in a cohort of Hispanic PD patients. Our results show that the frequency of certain NMS in our population is almost similar to the phenotype described in the global literature, but here we also provide data about the dynamics of NMS presentation in PD. Hence, our study adds valuable information useful to improve our understanding of NMS in PD, which may contribute to anticipate and individualize the diagnostic approach and therapeutic interventions among Hispanic PD patients.

## Materials and Methods

### Study Population

We conducted a cross-sectional study in a group of PD patients that were under regular follow-up at the outpatient clinic of the Department of Neurology of the Centro Medico Nacional “20 de Noviembre,” Instituto de Seguridad y Servicios Sociales de los Trabajadores del Estado (ISSSTE) in Mexico City. Patients attending to their follow-up medical appointments from January to December of 2015 that met the United Kingdom Parkinson's Disease Society Brain Bank (UKPDSBB) criteria for idiopathic PD were enrolled in the study. Individuals presenting with motor fluctuations during “off periods” were not eligible, as certain cognitive alterations that occur mainly during “off periods” could limit the patients' ability to report their symptoms (13). We also excluded subjects with parkinsonism due to alternative causes, as well as PD patients that underwent to surgical management and deep-brain stimulation for their motor symptoms, since these management strategies may affect the burden of NMS in PD (14). Moreover, patients unable to provide informed consent due to significant cognitive impairment were not considered for the study in order to exclude possible alterations in their capacity to self-report reliable information (3, 15). The current investigation was reviewed and approved by the Ethics committee of the Centro Médico Nacional “20 de Noviembre,” in Mexico City. All the enrolled individuals provided written informed consent to participate in the study.

### Clinical Evaluation and Data Retrieval

At the time of their recruitment, all participants were interviewed and examined neurologically by trained neurologists competent in the evaluation and management of movement disorders. Demographic (age and gender) and disease information (age at onset, disease duration, current use of medication) were obtained by direct interview or review of the clinical records of the patients. The progression of PD was evaluated according to the Hoehn and Yahr (H&Y) scale. The presence of NMS was assessed with the self-completed NMS questionnaire (NMSQuest) (16), which is a 30-items survey featuring “yes” and “no” response options that comprehensively screens the presence of the most relevant NMS in PD (16). For the current study, the items evaluated in such questionnaire were grouped based on the organization of NMS into domains according to the NMS scale (NMSS) (3). We excluded the item of legs swelling from the NMSQuest as it is not further considered within any of the NMSS domains. To address the dynamics of NMS presentation, participants were interrogated about the time of each NMS occurrence respect to the onset of their disease (defined as the time when motor symptoms appeared). For patients started complaining of new onset NMS at the moment of their recruitment, the time of NMS presentation was registered by the medical doctor as the interval between disease onset and the current date. In order to corroborate and match temporal information provided by participants during their interrogation, we performed a retrospective review of the medical registries of PD patients looking for previous NMSQuest evaluations or past medical notes describing the appearance of NMS symptoms. Patients that provided temporal information conflicting with the data retrieved from their medical records were considered only for the descriptive analysis of the frequency but not for the temporal pattern of NMS presentation.

### Statistical Analysis

Descriptive statistics were used to clinically characterize the study population. Frequencies and proportions were calculated for categorical data. Means, medians, standard deviations (SD), 95% confidence intervals (95% CI), and interquartile ranges (IQR) were used for continuous variables. The frequency of individual NMS was expressed as the percentage of patients presenting each item of the NMSQuest. In addition, when presenting the prevalence of NMS grouped as domains, we considered the proportion of patients presenting at least one item of each domain. Patients were divided into subgroups according to gender, age at disease onset, disease duration and HY stage. The analysis of differences in the frequency of individual NMS and NMS domains between patient subgroups was performed using the Chi-square or Fisher exact test. Differences in median times of NMS presentation respect to the onset of disease, as well as in other continuous variables between patient subgroups were estimated using the Mann-Whitney U or Student *T*-test as appropriate. For comparison of continuous variables between more than two subgroups of patients we used the Kruskal-Wallis test. Correlations between the total number of NMS per patient and the age at enrollment, age at disease onset or disease duration were estimated using the Pearson's and Spearman's rank correlation coefficients. Values of *p* < 0.05 were considered statistically significant. All analyses were conducted using IBM SPSS Statistics v20 (SPSS, Inc., Chicago, IL) and GraphPad Prism 8 (La Jolla, CA).

## Results

### Participants Characteristics

We enrolled a total of 120 PD patients. Their mean age at enrollment was 63.33 ± 8.60 years, whereas their mean age at onset of disease was 54.45 ± 10.32 years. From these 73.33% were males and 26.66% females with a mean age at disease onset of 53.64 ± 9.90 years and 56.69 ± 11.23 years, respectively (see Table 1). Thirty-five of the participants belong to the group of early-onset PD (EOPD) as their age at onset of disease was <50 years (17, 18), whereas 85 individuals were categorized as late-onset PD (LOPD) patients (>50 years). No statistically significant differences in the distribution of EOPD and LOPD cases were observed between males and females. The overall median time of disease duration from onset to recruitment was 8 years, but males had a significant longer duration of disease compared with females (Table 1). Most of the enrolled patients were at the H&Y stage 2, and no differences in the disease staging were observed between males and females. All the participants were under stable treatment with levodopa/carbidopa without any motor fluctuation at their recruitment. Demographic and disease characteristics of participants are summarized in Table 1.

**Table 1:** Participants characteristics.

**Variable**	**All** ***n* = 120**	**Males** ***n* = 88**	**Females** ***n* = 32**	***p*-value**
Gender				
Males, *n* (%)	88 (73.33)	NA	NA	NA
Females, *n* (%)	32 (26.66)			
Age (years), mean (SD)	63.33 (8.60)	63.05 (8.53)	64.13 (8.87)	0.5455
Age at onset (years), mean (SD)	54.45 (10.32)	53.64 (9.90)	56.69 (11.23)	0.1269
Age group				
EOPD, *n* (%)	35 (29.16)	27 (30.68)	8 (25)	0.5448
LOPD, *n* (%)	85 (70.83)	61 (69.31)	24 (75)	
Disease duration (years), median (IQR)	8 (5–12)	9 (5–12.75)	6 (3.25–10)	0.0466
Hoehn & Yahr stage, median (IQR)	2 (2–3)	2 (2–3)	2 (2–3)	0.7300
1, *n* (%)	11 (9.16)	6 (6.81)	5 (15.62)	0.1600
2, *n* (%)	63 (52.5)	49 (55.68)	14 (43.75)	0.3029
3, *n* (%)	33 (27.5)	23 (26.13)	10 (31.25)	0.6456
4, *n* (%)	12 (10)	9 (10.22)	3 (9.37)	>0.9999
5, *n* (%)	1 (0.83)	1 (1.13)	0 (0)	>0.9999

*EOPD, early-onset Parkinson's disease; IQR, 25%−75% interquartile range; LOPD, late-onset Parkinson's disease; NA, not applicable; NMS, non-motor symptom; SD, standard deviation. Differences in continuous variables between males and females were estimated using the Student-T test or Mann-Whitney U test. Differences in categorical variables were calculated using the Fisher's exact or Chi square test*.

### Gender Differences in the Frequency and Time of NMS Presentation in PD Patients

**Figure 1:**
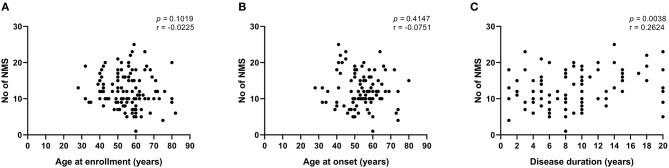
Correlations between disease characteristics and total number of NMS in PD patients. **(A)** Correlation between age at enrollment and number of NMS per patient. **(B)** Correlation between age at disease onset and number of NMS per patient. **(C)** Correlation between disease duration and number of NMS per patient. Correlations were estimated using the Pearson's **(A)** and Spearman's **(B,C)** rank correlation coefficients. NMS, non-motor symptoms.

All the participants enrolled in the current study presented at least 1 NMS. The median number of NMS per patient was 12, with no significant differences between males and females (Table 2). Interestingly, age at enrollment and age at disease onset, defined as the moment when cardinal motor symptoms manifested, did not correlate with the number of NMS per patient (Figures 1A,B), whereas disease duration showed a positive significant correlation with such variable (Figure 1C), suggesting that the burden of NMS in our population increased over time. When grouped as domains, the most frequent NMS observed in the study participants were miscellaneous (88.33%), followed by urinary tract (86.66%), sleep/fatigue (86.66%), gastrointestinal tract (85%), mood/cognition (77.5%), cardiovascular (75%), attention/memory (60%), sexual function (54.16%), and perceptual problems/hallucinations symptoms (40.83%, see Table 2). There were no significant differences between males and females in the frequency of any specific NMS domain. However, urinary tract, sleep/fatigue, attention/memory and sexual function NMS domains tended to be more common in males. The most frequent individual NMS were nocturia (73.33%), urinary urgency (70%), feeling sadness (60%), constipation (59.16%), insomnia (57.5%), drooling of saliva (50.83%), taste/smell impairment (55%), and hyperhidrosis (50%), as shown in Table 2. Only drooling of saliva and sexual dysfunction were significatively different between genders, as these symptoms occurred with a higher frequency in males. Memory impairment, nocturia, and loss of libido/hypersexuality also tended to be more common in males, whereas constipation, nausea/vomiting and feeling sadness were more frequent in females (Table 2).

**Table 2:** Frequency of NMS presentation in PD patients.

**NMS**	**Frequency**, ***n*** **(%)**			
	**All** **(*n* = 120)**	**Males** **(*n* = 88)**	**Females** **(*n* = 32)**	***p*-value**
No. of patients presenting at least 1 NMS	120 (100)	88 (100)	32 (100)	>0.9999
Number of NMS per patient, median (IQR)	12 (9–16)	12 (9–16.75)	12 (9.25–15)	0.5801
**Cardiovascular**	**90 (75)**	**65 (73.86)**	**25 (78.12)**	**0.8122**
Orthostatic lightheadedness/dizziness	59 (49.16)	43 (48.86)	16 (50)	>0.9999
Falls because of fainting	66 (55)	48 (54.54)	18 (56.25)	>0.9999
**Sleep/Fatigue**	**104 (86.66)**	**78 (88.63)**	**26 (81.25)**	**0.3627**
Excessive day time sleepiness	33 (27.5)	22 (25)	11 (34.37)	0.3575
Insomnia	69 (57.5)	49 (55.68)	20 (62.5)	0.5380
Intense/vivid dreams	47 (39.16)	38 (43.18)	9 (28.12)	0.1462
REM behavior disorder	53 (44.16)	42 (47.72)	11 (34.37)	0.2177
Restless legs	41 (34.16)	33 (37.5)	8 (25)	0.2768
**Mood/Cognition**	**93 (77.5)**	**67 (76.13)**	**26 (81.25)**	**0.6287**
Loss of interest	48 (40)	33 (37.5)	15 (46.87)	0.4025
Feeling sadness	72 (60)	49 (55.68)	23 (71.87)	0.1411
Anxiety	51 (42.5)	38 (43.18)	13 (40.62)	0.8375
**Perceptual problems/Hallucinations**	**49 (40.83)**	**34 (38.63)**	**15 (46.87)**	**0.5291**
Visual/auditory hallucinations	32 (26.66)	24 (27.27)	8 (25)	0.2800
Delusions	20 (16.66)	15 (17.04)	5 (15.62)	>0.9999
Diplopia	13 (10.83)	10 (11.36)	3 (9.37)	>0.9999
**Attention/Memory**	**72 (60)**	**55 (62.5)**	**17 (53.12)**	**0.4025**
Difficulties maintaining concentration	41 (34.16)	28 (31.81)	13 (40.62)	0.3904
Memory impairment	66 (55)	52 (59.09)	14 (43.75)	0.1511
**Gastrointestinal**	**102 (85)**	**75 (85.22)**	**27 (84.37)**	**>0.9999**
Drooling of saliva	61 (50.83)	51 (57.95)	10 (31.25)	0.0130
Difficulty in swallowing	46 (38.33)	36 (40.9)	10 (31.25)	0.3992
NAusea/vomiting	19 (15.83)	11 (12.5)	8 (25)	0.1543
Constipation	71 (59.16)	48 (54.54)	23 (71.87)	0.0976
Fecal incontinence	26 (21.66)	21 (23.86)	5 (15.62)	0.4540
Incomplete bowel emptying	43 (35.83)	31 (35.22)	12 (37.5)	0.8323
**Urinary**	**104 (86.66)**	**79 (89.77)**	**25 (78.12)**	**0.1281**
Urgency	84 (70)	64 (72.72)	20 (62.5)	0.3676
Nocturia	88 (73.33)	68 (77.27)	20 (62.5)	0.1600
**Sexual function**	**65 (54.16)**	**52 (59.09)**	**13 (40.62)**	**0.0974**
Loss of libido/hypersexuality	51 (42.5)	42 (47.72)	9 (28.12)	0.0627
Sexual dysfunction	55 (45.83)	46 (52.27)	9 (28.12)	0.0230
**Miscellaneous**	**106 (88.33)**	**76 (86.36)**	**30 (93.75)**	**0.3485**
Taste/smell impairment	66 (55)	47 (53.4)	19 (59.37)	0.6790
Weight fluctuations	55 (45.83)	40 (45.45)	15 (46.87)	>0.9999
Hyperhidrosis	60 (50)	44 (50)	16 (50)	>0.9999
Pain	49 (40.83)	35 (39.77)	14 (43.75)	0.8339

*IQR, 25%−75% interquartile range; NMS, non-motor symptom; PD, Parkinson's disease; REM, rapid eye movements. Differences in categorical variables between males and females were estimated using Fisher's exact or Chi square test. Differences in continuous variables between males and females were estimated using the Mann–Whitney U-test. Bold values indicate statistics for a global symptom domain/group, whereas unbold values correspond to each individual symptom included in a domain*.

**Figure 2:**
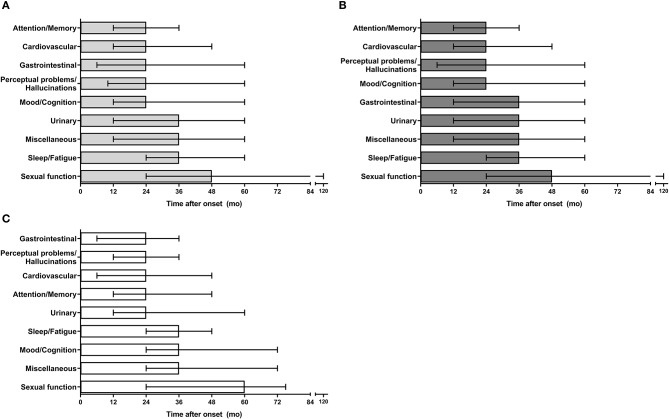
Time of NMS domains presentation in PD patients. **(A)** Temporal pattern of NMS domains presentation in all participants. **(B)** Time of NMS domains presentation in males. **(C)** Time of NMS domains presentation in females. mo, months; NMS, non-motor symptoms.

Any patient reported NMS before the onset of motor manifestations. NMS of the attention/memory domain where the earliest symptoms to occur during the course of PD, with a median interval from disease onset to symptom presentation of 24 months, whereas sexual function symptoms were the last to appear (see Figure 2A and Supplementary Table 1). Overall, the pattern of occurrence of NMS domains in our population from the earliest to the last was as follows: attention/memory, cardiovascular, gastrointestinal tract, perceptual problems/hallucinations, mood/cognition, urinary, miscellaneous, sleep/fatigue, and sexual function. In males, attention/memory, cardiovascular, perceptual problems/hallucinations, and mood/cognition were the earliest NMS domains to occur (Figure 2B), whereas gastrointestinal, perceptual problems/hallucinations, cardiovascular, attention/memory and urinary symptoms were the earliest NMS domains in females (Figure 2C). However, only gastrointestinal symptoms occurred significatively earlier in females (Supplementary Table 1).

**Figure 3:**
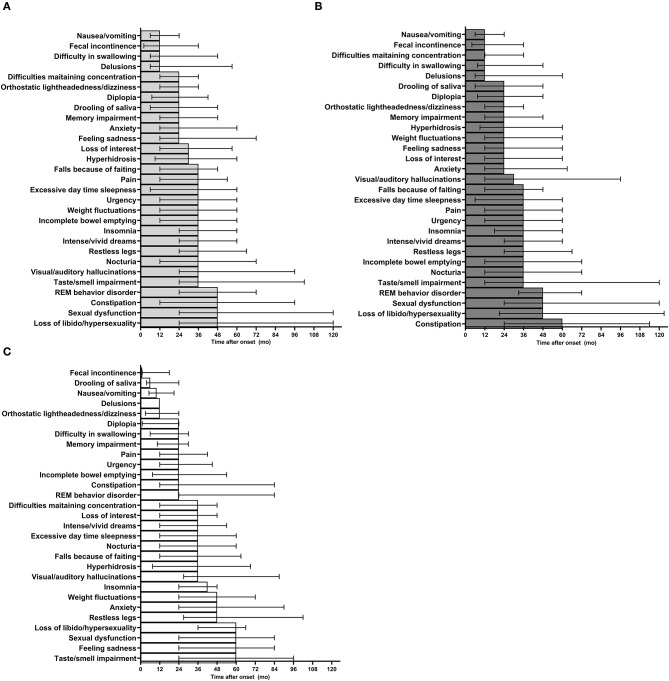
Temporal pattern of individual NMS presentation in PD patients. **(A)** Temporal pattern of individual NMS presentation in all participants. **(B)** Time of NMS presentation in males. **(C)** Time of NMS presentation in females. mo, months; NMS, non-motor symptoms; REM, rapid-eye movements.

The dynamics of individual NMS in PD showed that nausea/vomiting was the earliest symptom observed in all patients independently of their gender, with a median time of presentation of 12 months. This symptom was followed by fecal incontinence, difficulty in swallowing, and delusions, whereas sexual dysfunction and changes in interest for sex were the last symptoms to occur (Figure 3A and Supplementary Table 1). Among males, the earliest NMS reported by participants were nausea/vomiting, fecal incontinence, difficulties maintaining concentration, difficulty in swallowing and delusions, whereas constipation was the last symptom to appear (Figure 3B). On the other hand, fecal incontinence, drooling of saliva, nausea/vomiting, and delusions were the earliest individual NMS in females, whereas loss of libido/hypersexuality, sexual dysfunction, feeling sadness and taste/smell impairment occurred late during the course of PD in the same group (Figure 3C). Despite these gender patterns of NMS presentation, only drooling of saliva occurred significatively earlier in females than males (Supplementary Table 1).

### Frequency of NMS in PD Patients According to Their Age at Disease Onset and Duration of Disease

Our previous results suggested that the burden of NMS is not influenced by the age at onset of PD in our population (Figure 1B). Conversely, recent studies have shown that the frequency of NMS is different among EOPD and LOPD individuals (9, 19). Hence, we further looked for differences in the burden of NMS between patients grouped according to their age at onset of disease. In the current study, the median age at onset of disease in EOPD patients was 43 years, whereas median age at onset of LOPD participants was 58 years (Table 3). Disease duration was significatively longer in EOPD than LOPD patients (10 vs. 8 years, *p* = 0.0070). However, we found that the total number of NMS per patient did not differ between participants grouped according to their age at disease onset (Table 3). In fact, only miscellaneous and gastrointestinal NMS domains tended to occur more frequently among LOPD without reaching statistical significance. Meanwhile, orthostatic lightheadedness/dizziness was the only individual NMS that occurred with a significant higher frequency in LOPD as compared with EOPD, whereas falls because of fainting were significatively more common in EOPD patients (Table 3).

**Table 3:** Frequency of NMS presentation in PD patients according to age at disease onset.

**Variable/NMS**	**Frequency**, ***n*** **(%)**		
	**EOPD** **(*n* = 35)**	**LOPD** **(*n* = 85)**	***p*-value**
Age at onset (years), median (IQR)	43 (40–47)	58 (54–63.5)	<0.0001
Disease duration (years), median (IQR)	10 (6–15)	8 (5–10)	0.0070
Hoehn & Yahr stage, median (IQR)	2 (2–3)	2 (2–3)	0.0366
1, *n* (%)	1 (2.85)	10 (11.76)	0.1726
2, *n* (%)	17 (48.57)	46 (54.11)	0.6882
3, *n* (%)	10 (28.57)	23 (27.05)	>0.9999
4, *n* (%)	6 (17.14)	6 (7.05)	0.1060
5, *n* (%)	1 (2.85)	0 (0)	0.2917
Number of NMS per patient, median (IRQ)	12 (10–16)	12 (8–18)	0.9827
**Cardiovascular**	**26 (74.28)**	**64 (75.29)**	**>0.9999**
Orthostatic lightheadedness/dizziness	11 (31.42)	48 (56.47)	0.0160
Falls because of fainting	25 (71.42)	41 (48.23)	0.0263
**Sleep/Fatigue**	**28 (80)**	**76 (89.41)**	**0.2356**
Excessive day time sleepiness	14 (40)	19 (22.35)	0.0710
Insomnia	21 (60)	48 (56.47)	0.8396
Intense/vivid dreams	13 (37.14)	34 (40)	0.8387
REM behavior disorder	15 (42.85)	38 (44.70)	>0.9999
Restless legs	11 (31.42)	30 (35.29)	0.8327
**Mood/Cognition**	**29 (82.85)**	**64 (75.29)**	**0.4733**
Loss of interest	16 (45.71)	32 (37.64)	0.4209
Feeling sadness	20 (57.14)	52 (61.17)	0.6874
Anxiety	18 (51.42)	34 (40)	0.3118
**Perceptual problems/Hallucinations**	**17 (48.57)**	**32 (37.64)**	**0.3098**
Visual/auditory hallucinations	13 (37.14)	19 (22.35)	0.1142
Delusions	6 (17.14)	14 (16.47)	>0.9999
Diplopia	5 (14.28)	8 (9.41)	0.5200
**Attention/Memory**	**21 (60)**	**51 (60)**	**>0.9999**
Difficulties maintaining concentration	14 (40)	27 (31.76)	0.4041
Memory impairment	20 (57.14)	46 (54.11)	0.8412
**Gastrointestinal**	**28 (80)**	**77 (90.58)**	**0.1329**
Drooling of saliva	14 (40)	47 (55.29)	0.1607
Difficulty in swallowing	13 (37.14)	33 (38.82)	>0.9999
NAusea/vomiting	3 (8.57)	16 (18.82)	0.2700
Constipation	20 (57.14)	51 (60)	0.8392
Fecal incontinence	7 (20)	19 (22.35)	>0.9999
Incomplete bowel emptying	15 (42.85)	28 (32.94)	0.4024
**Urinary**	**31 (88.57)**	**73 (85.88)**	**0.7769**
Urgency	24 (68.57)	60 (70.58)	0.8296
Nocturia	25 (71.42)	63 (74.11)	0.8216
**Sexual function**	**22 (62.85)**	**43 (50.58)**	**0.2345**
Loss of libido/hypersexuality	18 (51.42)	33 (38.82)	0.2275
Sexual dysfunction	20 (57.14)	35 (41.17)	0.1578
**Miscellaneous**	**28 (80)**	**78 (91.76)**	**0.1133**
Taste/smell impairment	20 (57.14)	46 (54.11)	0.8412
Weight fluctuations	16 (45.71)	39 (45.88)	>0.9999
Hyperhidrosis	21 (60)	39 (45.88)	0.2280
Pain	14 (40)	35 (41.17)	>0.9999

*EOPD, early-onset Parkinson's disease; IQR, 25%−75% interquartile range; LOPD, late-onset Parkinson's disease; NMS, non-motor symptom; PD, Parkinson's disease; REM, rapid eye movements. Differences between males and females were estimated using the Fisher's exact or Chi square test. Differences in continuous variables were estimated using the Mann-Whitney U test. Bold values indicate statistics for a global symptom domain/group, whereas unbold values correspond to each individual symptom included in a domain*.

On the other hand, we also grouped study participants according to their duration of disease (<10 years vs. > 10 years) and estimated differences in the frequency of NMS. The median age at onset of disease was significatively higher in the group of patients with a shorter duration of disease (Table 4). Interestingly, patients with a duration of disease >10 years had a higher number of NMS per individual as compared with the group of participants with a duration of disease < 10 years (13 vs. 10 symptoms per patient, *p* = 0.0098; Table 4). In general, all the NMS domains except sleep/fatigue tended to occur with a higher frequency in participants with a longer duration of disease. Nonetheless, only mood/cognition, sexual function and urinary symptoms were significatively more common in patients with a duration of disease > 10 years. Our analysis of individual NMS showed that urinary urgency, constipation, feeling sadness, sexual dysfunction, REM behavior disorder, loss of interest, anxiety, and incomplete bowel emptying occurred with a significant higher frequency in the group of participants with a longer duration of disease (Table 4).

**Table 4:** Frequency of NMS presentation in PD patients according to disease duration.

**Variable/NMS**	**Frequency**, ***n*** **(%)**		
	**<10 years** **(*n* = 65)**	**>10 years** **(*n* = 55)**	***p*-value**
Age at onset (years), median (IQR)	57 (50–65)	51 (42–58)	<0.0001
Number of NMS per patient, median (IQR)	10 (8.5–15)	13 (10–18)	0.0098
**Cardiovascular**	**46 (70.76)**	**44 (80)**	**0.2931**
Orthostatic lightheadedness/dizziness	33 (50.76)	26 (47.27)	0.7180
Falls because of fainting	32 (49.23)	34 (61.81)	0.1991
**Sleep/Fatigue**	**57 (87.69)**	**47 (85.45)**	**0.7910**
Excessive day time sleepiness	14 (21.53)	19 (34.54)	0.1509
Insomnia	40 (61.53)	29 (52.72)	0.3588
Intense/vivid dreams	26 (40)	21 (38.18)	0.8535
REM behavior disorder	22 (33.84)	31 (56.36)	0.0167
Restless legs	19 (29.23)	22 (40)	0.2492
**Mood/Cognition**	**45 (69.23)**	**48 (87.27)**	**0.0273**
Loss of interest	19 (29.23)	29 (52.72)	0.0147
Feeling sadness	33 (50.76)	39 (70.90)	0.0269
Anxiety	22 (33.84)	29 (52.72)	0.0430
**Perceptual problems/Hallucinations**	**26 (40)**	**23 (41.81)**	**0.8543**
Visual/auditory hallucinations	16 (24.61)	16 (29.09)	0.6796
Delusions	13 (20)	7 (12.72)	0.3328
Diplopia	7 (10.76)	6 (10.90)	0.9999
**Attention/Memory**	**36 (55.38)**	**36 (65.45)**	**0.3499**
Difficulties maintaining concentration	20 (30.76)	21 (38.18)	0.4426
Memory impairment	32 (49.23)	34 (61.81)	0.1991
**Gastrointestinal**	**56 (86.15)**	**49 (89.09)**	**0.7835**
Drooling of saliva	31 (47.69)	30 (54.54)	0.4700
Difficulty in swallowing	30 (46.15)	16 (29.09)	0.0621
NAusea/vomiting	11 (16.92)	8 (14.54)	0.8049
Constipation	32 (49.23)	39 (70.90)	0.0249
Fecal incontinence	14 (21.53)	12 (21.81)	0.9999
Incomplete bowel emptying	16 (24.61)	27 (49.09)	0.0073
**Urinary**	**51 (78.46)**	**53 (96.36)**	**0.0057**
Urgency	38 (58.46)	46 (83.63)	0.0029
Nocturia	44 (67.69)	44 (80)	0.1504
**Sexual function**	**29 (44.61)**	**36 (65.45)**	**0.0278**
Loss of libido/hypersexuality	25 (38.46)	26 (47.27)	0.3588
Sexual dysfunction	22 (33.84)	33 (60)	0.0057
**Miscellaneous**	**57 (87.69)**	**49 (89.09)**	**0.9999**
Taste/smell impairment	34 (52.30)	32 (58.18)	0.5825
Weight fluctuations	33 (50.76)	22 (40)	0.2727
Hyperhidrosis	29 (44.61)	31 (56.36)	0.2716
Pain	27 (41.53)	22 (40)	0.9999

*IQR, 25%−75% interquartile range; NMS, non-motor symptom; PD, Parkinson's disease; REM, rapid eye movements. Differences in frequencies between subgroups were estimated using the Fisher exact or Chi square test. Differences in continuous variables were estimated using the Mann-Whitney U test. Bold values indicate statistics for a global symptom domain/group, whereas unbold values correspond to each individual symptom included in a domain*.

### NMS Presentation According to Disease Stage

Previous studies have shown that the severity of motor findings and NMS in PD patients are closely related (5, 11, 19), suggesting that the mechanisms of cellular degeneration underlying this disease parallelly affect multiple neuronal systems over time. Hence, in order to determine differences in the frequency of NMS presentation according to the degree of motor dysfunction, here we also grouped study participants according to their HY stage. As expected, we found that age at onset was higher in patients presenting at initial stages of disease, and disease duration was significatively longer in those presenting at late disease stages (see Table 5). Furthermore, we observed that the total number of NMS per patient increased as the HY staging progressed. Despite most patients were at HY2 stage at the moment of their enrollment, the proportion of patients presenting symptoms of the gastrointestinal tract, urinary tract, mood/cognition, cardiovascular, and sexual function NMS domains was higher in the HY 4–5 group (Table 5). Interestingly, sleep/fatigue and attention/memory symptoms were more frequent at HY1 stage. Only difficulty in swallowing and loss of libido/hypersexuality were significatively more frequent at advanced stages of disease (HY 4–5), whereas feeling sadness was most common at HY 3 stage (Table 5).

**Table 5:** Frequency of NMS in PD patients according to the stage of disease.

**Variable/NMS**	**Frequency**, ***n*** **(%)**				
	**HY 1** **(*n* = 11)**	**HY 2** **(*n* = 63)**	**HY 3** **(*n* = 33)**	**HY 4/5** **(*n* = 13)**	***p*-value**
Age at onset (years), median (IQR)	60 (55–64)	54 (49–60)	55 (47–60)	42 (40.5–56)	0.0282
Disease duration (years), median (IQR)	6 (1–8)	8 (4–10)	10 (8.5–13.5)	15 (11–17)	<0.0001
Number of NMS per patient, median (IQR)	10 (5–13)	10 (9–16)	12 (10–16)	15 (9.5–17.5)	0.2521
**Cardiovascular**	**7 (63.63)**	**45 (71.42)**	**27 (81.81)**	**11 (84.61)**	**0.4496**
Orthostatic lightheadedness/dizziness	4 (36.36)	31 (49.20)	19 (57.57)	5 (38.46)	0.5219
Falls because of fainting	4 (36.36)	36 (57.14)	16 (48.48)	10 (76.92)	0.1910
**Sleep/Fatigue**	**11 (100)**	**55 (87.30)**	**28 (84.84)**	**10 (76.92)**	**0.4110**
Excessive day time sleepiness	1 (9.09)	18 (28.57)	11 (33.33)	3 (23.07)	0.4580
Insomnia	8 (72.72)	33 (52.38)	19 (57.57)	9 (69.23)	0.4841
Intense/vivid dreams	1 (9.09)	30 (47.61)	13 (39.39)	3 (23.07)	0.0581
REM behavior disorder	4 (36.36)	29 (46.03)	14 (42.42)	6 (46.15)	0.9357
Restless legs	2 (18.18)	21 (33.33)	15 (45.45)	3 (23.07)	0.2782
**Mood/Cognition**	**4 (36.36)**	**49 (77.77)**	**28 (84.84)**	**12 (92.30)**	**0.0040**
Loss of interest	2 (18.18)	28 (44.44)	11 (33.33)	7 (53.84)	0.2261
Feeling sadness	3 (27.27)	34 (53.96)	27 (81.81)	8 (61.53)	0.0061
Anxiety	3 (27.27)	26 (41.26)	13 (39.39)	9 (69.23)	0.1708
**Perceptual problems/Hallucinations**	**6 (54.54)**	**21 (33.33)**	**17 (51.51)**	**5 (38.46)**	**0.2712**
Visual/auditory hallucinations	4 (36.36)	15 (23.80)	8 (24.24)	5 (38.46)	0.6115
Delusions	1 (9.09)	12 (19.04)	5 (15.15)	2 (15.38)	0.8539
Diplopia	2 (18.18)	4 (6.34)	7 (21.21)	0 (0)	0.0662
**Attention/Memory**	**8 (72.72)**	**37 (58.73)**	**19 (57.57)**	**8 (61.53)**	**0.8306**
Difficulties maintaining concentration	4 (36.36)	20 (31.74)	12 (36.36)	5 (38.46)	0.9473
Memory impairment	7 (63.63)	33 (52.38)	18 (54.54)	8 (61.53)	0.8653
**Gastrointestinal**	**10 (90.90)**	**56 (88.88)**	**26 (78.78)**	**13 (100)**	**0.2237**
Drooling of saliva	4 (36.36)	31 (49.20)	20 (60.60)	6 (46.15)	0.5005
Difficulty in swallowing	2 (18.18)	30 (47.61)	7 (21.21)	7 (53.84)	0.0223
NAusea/vomiting	3 (27.27)	8 (12.69)	7 (21.21)	1 (7.69)	0.4061
Constipation	8 (72.72)	35 (55.55)	18 (54.54)	10 (76.92)	0.3668
Fecal incontinence	3 (27.27)	12 (19.04)	9 (27.27)	2 (15.38)	0.7122
Incomplete bowel emptying	4 (36.36)	21 (33.33)	13 (39.39)	5 (38.46)	0.9416
**Urinary**	**9 (81.81)**	**56 (88.88)**	**26 (78.78)**	**13 (100)**	**0.2342**
Urgency	6 (54.54)	44 (69.84)	23 (69.69)	11 (84.61)	0.4618
Nocturia	9 (81.81)	48 (76.19)	23 (69.69)	8 (61.53)	0.6115
**Sexual function**	**6 (54.54)**	**31 (49.20)**	**17 (51.51)**	**11 (84.61)**	**0.1343**
Loss of libido/hypersexuality	6 (54.54)	24 (38.09)	11 (33.33)	10 (76.92)	0.0352
Sexual dysfunction	3 (27.27)	26 (41.26)	16 (48.48)	10 (76.92)	0.0655
**Miscellaneous**	**9 (81.81)**	**55 (87.30)**	**31 (3.03)**	**11 (84.61)**	**0.6372**
Taste/smell impairment	7 (63.63)	30 (47.61)	22 (66.66)	7 (53.84)	0.3156
Weight fluctuations	2 (18.18)	33 (52.38)	16 (48.48)	4 (30.76)	0.1240
Hyperhidrosis	4 (36.36)	30 (47.61)	17 (51.51)	9 (69.23)	0.4050
Pain	3 (27.27)	22 (34.92)	17 (51.51)	7 (53.84)	0.2388

*IQR, 25%−75% interquartile range; NMS, non-motor symptom; PD, Parkinson's disease; REM, rapid eye movements. Differences in frequencies of NMS between disease stages were estimated using the Chi square test. Differences in continuous variables between subgroups were analyzed using the Kruskal-Wallis test. Bold values indicate statistics for a global symptom domain/group, whereas unbold values correspond to each individual symptom included in a domain*.

## Discussion

It has been increasingly recognized that the clinical phenotype of PD is greatly heterogeneous, encompassing both the presence of cardinal motor symptoms, as well as the progressive development of a wide range of NMS, which in conjunct exert a detrimental impact on the functional independence of ill subjects. In fact, it is now accepted that NMS are more common among PD patients compared to age- and gender-matched healthy individuals (8, 10, 20, 21). Moreover, it has been described that NMS can independently impact on the QoL of patients with PD (4, 5), contributing even more than motor symptoms to the clinical burden of the disease (3, 4). However, NMS remained unappreciated for a long time hindering the implementation of individualized therapeutic strategies to limit the clinical implications of specific manifestations. In this regard, the incomplete understanding of the frequency and temporal patterns of NMS presentation along the progression of disease has impeded clinicians to anticipate their occurrence in order to better rationalize and plan their management. Currently, it is known that some NMS can even precede the appearance of cardinal symptoms (22, 23), but the time at which other NMS occur with respect to the beginning of the motor abnormalities has not been well-described. Also, more studies addressing the frequency and pattern of NMS presentation among different populations are needed to understand how these symptoms affect PD patients according to their ethnic background. This is quite important as emerging evidence suggests that the clinical phenotype of PD may vary between distinct populations probably because of the effect of genetic, sociocultural, sociodemographic and environmental factors specific of each ethnic group (12). Despite these, the burden of NMS in PD has mainly been explored in white-Caucasian populations.

Here, we investigated the frequency and dynamics of NMS presentation in a group of non-Caucasian Hispanic patients with PD. To our knowledge, the current study is one of the largest investigations about NMS in PD conducted in Latin American populations, and one of the first reports describing the temporal pattern of NMS occurrence with respect to the onset of motor manifestations. Our results showed, firstly, that most of the clinical and demographic characteristics of our study participants, such as their age at disease onset and disease duration, are similar to the clinical features described in other reports about the clinical phenotype of PD in Mexicans (24–26). Nonetheless, we found a lower proportion of females affected by PD, which may be accounted to discrepancies in study designs (single-center vs. multi-center) and gender differences in the incidence and access to health care of PD patients across different regions of our country. Secondly, we found that 100% of our patients reported the presence of at least 1 NMS, which coincides with the results of other studies showing a high frequency of non-motor manifestations in individuals with PD from Italy and Morocco (11, 27). In fact, we also observed that the median number of NMS per patient in our study was 12, similar to other large studies conducted in Mexico, Italy and the United Kingdom that have also showed that the number of NMS affecting each patient ranges from 8 to 11 (7, 11, 25). Collectively, these data reveal that the burden of NMS in PD, in terms of the number of symptoms per patient, is high even among populations with different ethnic background.

Although the frequency of NMS in our population was similar to other groups, our results also demonstrate that the effect of certain demographic characteristics on the burden of NMS in PD may not be the same among different populations. For instance, several studies conducted in Asia have shown that females present a higher number of NMS compared to males (19, 28). Conversely, we did not find gender differences in the number of individuals reporting NMS nor in the amount of NMS per patient. Perhaps, the lower proportion of females enrolled in the current study, as well as they lower duration of disease compared to males explain why we did not find gender differences in the burden of NMS. Therefore, although other studies addressing NMS in PD have also reported a higher proportion of male participants (29), the gender comparisons described here must be interpreted taking in consideration possible bias induced by a lower number of females enrolled in our study. Moreover, contrary to other investigations in Chinese and Serbian patients with PD, we did not find significant differences in the number of NMS per individual between EOPD and LOPD groups (9, 19), which may be explained by discrepancies in cut-off values used in different studies to classify patients according to their age at disease onset.

On the other hand, our findings together with the results of previous studies suggest that although the burden of NMS in terms of number of symptoms per patient may vary according to certain demographic characteristics, the frequency of some NMS domains is similar across several populations. In this regard, we observed that the most common NMS domains in our study were miscellaneous, urinary tract, sleep/fatigue, gastrointestinal, mood/cognition and cardiovascular NMS domains. Similar findings were reported in two studies conducted in Mexico City and Monterrey, where the investigators observed that miscellaneous, urinary tract, sleep/fatigue and mood/cognition domains are very common in Mexican patients with PD (25, 26). Moreover, it has been found that sleep/fatigue, mood/cognition, and miscellaneous are the most frequent NMS domains in Chinese individuals with PD (19), whereas sleep/fatigue, urinary tract and gastrointestinal domains are more common in Moroccans (27). Interestingly, some of these are among the NMS domains that better discriminate between PD and normal elderly (20, 21). Thus, these data suggest that the mechanisms responsible of the occurrence of NMS in PD may be conserved across populations, and despite the multisystem nature of the disease, certain functional domains of the central nervous system might be more susceptible to get affected. In fact, a recent work showed almost similar phenotypes of NMS domain predominance in PD patients from different ethnic groups (29), supporting our previous assumption. Despite this, we do not rule out the possibility that genetic ancestry may drive subtle differences in the prevalence of certain NMS domains.

In the current study, the most frequent individual NMS were nocturia, urgency, feeling sadness, constipation, insomnia, and drooling of saliva. Other reports have also shown that the most common NMS in British PD patients are urinary (7). Similarly, nocturia, urgency and drooling of saliva were the most frequent NMS in a multicenter study conducted in Spain and the United Kingdom (4). Among males, we found that the most common NMS domains were urinary, sleep/fatigue, attention/memory, and sexual function, and the most common individual symptoms were drooling of saliva and sexual dysfunction. In females, miscellaneous, gastrointestinal, and mood/cognition were the most frequent NMS domains, whereas constipation, nausea/vomiting, and feeling sadness were the most common individual symptoms. These gender phenotypes have also been observed in other studies conducted in European populations (5). Of note, several research groups including us, have also found that females tend to present mood alterations with higher frequency than males (5, 19, 28), and depression can even precede the beginning of motor symptoms in females (23). A possible explanation for this gender difference in the presentation of depression may be related to the higher burden of NMS that has been shown among females in other studies (19, 28), and in fact, a higher burden of NMS has been associated with increased prevalence of depression in general (30). Also, in a previous study in Mexican PD patients, it was found that females were more propense to be depressed and their Unified Parkinson's Disease Rating Scale part III (UPDRS-III) scores were higher compared to males (30). Hence, it is possible that both motor and non-motor manifestations exert a higher impact on the mood of female PD patients. However, here we did not find gender differences in the frequency of NMS nor in the severity of PD according to the H&Y scale, thus we propose that the higher incidence of depression in females may be associated with their degree of self-awareness of motor and non-motor manifestations, or to other sociocultural factors.

Taken together, our results and data provided by other studies remark the necessity to focus more therapeutic strategies on the management of NMS in PD. Specifically, greater attention must be pay to miscellaneous, urinary tract, sleep/fatigue, gastrointestinal and mood/cognition NMS domains due to their high frequency, as well as to those symptoms that have demonstrated a higher impact on the QoL in PD, such as depression, gastrointestinal symptoms, and sleep disorders (7, 10). In this manner, an integral treatment approach focused both on motor manifestations and NMS can improve the welfare of patients living with PD. Furthermore, being able to anticipate the occurrence of NMS would be another strategy of great benefit for the management of PD. However, there is not enough information about the temporal pattern of NMS presentation during the course of the disease among different populations of PD patients. Currently, depression, sleep disorders, constipation and anxiety have been shown to occur early before motor symptom onset in Mexicans with PD (23). Constipation and incomplete bowel emptying also precede motor symptoms in Argentinians (22). In this context, the current study adds valuable evidence about the time at which different NMS occur with respect to the onset of motor symptoms. Regarding temporal presentation of NMS grouped by domains, we found that attention/memory was the earliest domain to occur in all the participants as well as among males. This is not a surprising finding, as it has been widely recognized that PD patients can present a certain degree of cognitive decline early during the course of their disease (31, 32), mostly in the form of mild cognitive impairment that can progress to PD dementia over time. Although the mechanisms associated with cognitive disfunction in PD are not well-understood, evidence suggests a mixture of Lewy bodies and Alzheimer pathologic mechanisms (33–35). Thus, our data further justify the early performance of neuropsychological evaluations looking for the presence of memory impairment or attention deficits to timely recognize and treat these symptoms. Our analysis of the temporal presentation of individual NMS also support an early screening for gastrointestinal manifestations, as some of these symptoms, such as nausea/vomiting, fecal incontinence and drooling of saliva occurred early both in male and female patients, and as mentioned before, gastrointestinal symptoms are among the NMS that impact more on the QoL of individuals with PD (7, 10). Interestingly, among the early gastrointestinal symptoms described here, the premature occurrence of fecal incontinence in some of our patients is quite unusual. Even when it is well-accepted that bowel disfunction is common in PD (36), this is mostly manifested as constipation instead of incontinence. In fact, fecal incontinence has not been observed with higher frequency among individuals with PD compared to healthy people (37). Therefore, as only 28 out of our 120 enrolled individuals reported such symptom, it is possible that this phenomenon was related to normal aging or to other possible nutritional, gastrointestinal or neurological conditions not recognized in our study. Another remarkable finding was that sexual function items were the less frequent and the last NMS observed in our population. This finding can be interpreted as a low incidence of sexual function problems among PD patients, or it may be related with differences in the patients' confidence to report such manifestations in the self-completed NMSQuest. Indeed, Rodriguez-Violante and colleagues found that the sexual function items are not answered by some patients unless direct interrogation is performed (25). Therefore, the frequency of sexual function symptoms in PD may be higher than expected.

Finally, we further confirm that the frequency and number of NMS per patient positively correlates with disease duration and severity of motor symptoms, as previously demonstrated by other investigators (5, 11, 19). We showed that gastrointestinal, urinary, mood/cognition, cardiovascular and sexual function NMS are more frequent in H&Y 4–5. These findings are very similar to data reported by Kadastik-Eerme and colleagues who showed that symptoms of the mood/cognition, sleep/fatigue and urinary domains are more frequent at later H&Y stages in PD patients from Estonia (5). Muller and colleagues also showed that a higher H&Y stage was associated with increased frequency of drooling of saliva, constipation, increased sweating and difficulties in swallowing among Norwegians (8). Moreover, in another study conducted in Serbians with PD, H&Y stage was a predictor of higher number of NMS (9). Collectively, these findings indicate that PD patients will require a more complex and multi-disciplinary management as their disease progresses, remarking the importance of the early detection and treatment of some NMS according to the time at which they occur, in order to plan and administrate different therapeutic strategies at different moments during the course of the disease.

The current study has some limitations that must be taken into consideration when interpreting our findings. On the one hand, we used the self-completed NMSQuest to evaluate the frequency of individual NMS, which is answered by the patient, instead of the NMSS that is applied and filled by the clinical neurologist and provides additional data about the severity of NMS. Therefore, although all of our study participants were able to provide informed consent, which may indicate a sufficient cognitive function (3, 15), we could not rule out possible bias in our estimates of the frequency of NMS induced by differences in their degree of self-awareness and performance in answering the NMSQuest. Notwithstanding, it is important to acknowledge that this bias may occur by using the NMSQuest and NMSS, as these screening tools require that patients understand the items of both questionnaires (3, 16). Indeed, there is an extra source of possible inaccuracy in the evaluation of NMS when using the NMSS, as the results also depend on the ability of physicians to appropriately interrogate the presence of NMS and objectively evaluate the severity of each item (3). Furthermore, previous data suggest that the results of NMSS highly coincide with those obtained by the NMSQuest (3), and initial studies validating these tools have demonstrated their applicability to the wide range of PD patients, among which a proportion of individuals with cognitive decline it is expected to be observed, with the only exception of those with established dementia (3, 16). Despite this, it would have been important to describe the cognitive status of our study population using an objective neuropsychological test, as well as to provide a better description of their education level to better correlate and support the NMS phenotypes observed here. On the other hand, we did not evaluate the QoL of our study participants using quantitative scales, thus we were not able to evaluate the impact of the frequency and burden of NMS on the welfare of PD patients. However, the detrimental impact of NMS on the QoL of individuals with this neurodegenerative disorder has been well-described in previous reports (3–5), whereas the main goal of the current study was to provide a better description of the frequency and temporal pattern of presentation of NMS in Hispanic PD, which is a population not usually studied. Finally, a relevant limitation of the current study is that our description of the temporal presentation of NMS in PD was constructed based on pooled cross-sectional data. Therefore, future prospective studies are needed to corroborate the findings presented here.

Despite these limitations, our study represents one of the few clinical descriptions of the frequency and temporal pattern of NMS presentation in Hispanic PD patients available in the literature. Hence, our study may provide data of great utility to improve our understanding of NMS in PD, which may contribute to anticipate and individualize the diagnostic approach and therapeutic interventions among different populations of PD patients.

## Data Availability Statement

The datasets generated for this study are available on request to the corresponding author.

## Ethics Statement

The studies involving human participants were reviewed and approved by Ethics committee of the Centro Medico Nacional 20 de Noviembre, ISSSTE, Mexico City, Mexico. The patients provided their written informed consent to participate in this study.

## Author Contributions

CS-M designed the study, examined, followed PD patients, and retrieved clinical data. LN-O and NP-Á followed PD patients, acquired clinical data, and revised the manuscript for intellectual content. JC-P designed the study, analyzed the data, and drafted the manuscript. PG-O contributed in the writing process of the manuscript and revised it for intellectual content. All the authors approved the final version of the manuscript.

### Conflict of Interest

The authors declare that the research was conducted in the absence of any commercial or financial relationships that could be construed as a potential conflict of interest.
