# Supplementary material for: Frequency and Dynamics of Non-motor Symptoms Presentation in Hispanic Patients With Parkinson Disease
Source: Front Neurol. 2019 Nov 14;10:1197. doi: 10.3389/fneur.2019.01197 (PMC6868116; doi:10.3389/fneur.2019.01197)
Supplement: Supplementary file 1 [file Table_1.docx]

| **Supplementary Table 1. Dynamics of NMS presentation in PD patients.** | | | | |
| --- | --- | --- | --- | --- |
| **NMS** | **Time of presentation after onset (mo), median (IQR)** | | | |
|  | **All** | **Males** | **Females** | ***p* value** |
| **Cardiovascular**  Orthostatic lightheadedness/dizziness  Falls because of fainting | **24.00 (12.00 – 48.00)**  24.00(12.00 – 36.00)  36.00(12.00 – 48.00) | **24.00 (12.00 – 48.00)**  24.00(12.00 – 36.00)  36.00(12.00 – 48.00) | **24.00 (6.00 – 48.00)**  12.00(3.00 – 24.00)  36.00(12.00 – 63.00) | **0.2889**  0.0932  0.3744 |
| **Sleep/Fatigue**  Excessive day time sleepiness  Insomnia  Intense/vivid dreams  REM behavior disorder  Restless legs | **36.00(24.00 – 60.00)**  36.00(6.00 – 60.00)  36.00(24.00 – 60.00)  36.00(24.00 – 60.00)  36.00(24.00 – 72.00)  36.00(24.00 – 66.00) | **36.00(24.00 – 60.00)**  36.00(6.00 – 60.00)  36.00(18.00 – 60.00)  36.00(24.00 – 60.00)  48.00(33.00 – 72.00)  36.00(24.00 – 66.00) | **36.00(24.00 – 48.00)**  36.00(12.00 – 60.00)  42.00(24.00 – 48.00)  36.00(12.00 – 54.00)  24.00(24.00 – 84.00)  48.00(27.00 – 102.0) | **0.7428**  0.5540  0.7784  0.9414  0.2025  0.4676 |
| **Mood/Cognition**  Loss of interest  Feeling sadness  Anxiety | **24.00 (12.00 – 60.00)**  30.00(12.00 – 57.00)  24.00 (12.00 – 72.00)  24.00 (12.00 – 60.00) | **24.00 (12.00 – 60.00)**  24.00 (12.00 – 60.00)  24.00 (12.00 – 60.00)  24.00 (12.00 – 63.00) | **36.00(24.00 – 72.00)**  36.00(12.00 – 48.00)  60.00(24.00 – 84.00)  48.00(24.00 – 90.00) | **0.0721**  0.8626  0.1087  0.2273 |
| **Perceptual problems/Hallucinations**  Visual/auditory hallucinations  Delusions  Diplopia | **24.00 (10.00 – 60.00)**  36.00 (24.00 – 96.00)  12.00 (6.00 – 57.00)  24.00 (7.00 – 42.00) | **24.00 (6.00 – 60.00)**  30.00(12.00 – 96.00)  12.00 (6.00 – 60.00)  24.00 (7.50 – 48.00) | **24.00(12.00 – 36.00)**  36.00(27.00 – 87.00)  12.00(12.00 – 12.00)  24.00 (1.00 – 24.00) | **0.9063**  0.5370  >0.9999  0.5070 |
| **Attention/Memory**  Difficulties maintaining concentration  Memory impairment | **24.00(12.00 – 36.00)**  24.00 (12.00 – 36.00)  24.00 (12.00 – 48.00) | **24.00(12.00 – 36.00)**  12.00 (12.00 – 36.00)  24.00 (12.00 – 48.00) | **24.00(12.00 – 48.00)**  36.00(12.00 – 48.00)  24.00(10.50 – 30.00) | **0.8947**  0.2419  0.3097 |
| **Gastrointestinal**  Drooling of saliva  Difficulty in swallowing  Nausea/vomiting  Constipation  Fecal incontinence  Incomplete bowel emptying | **24.00 (6.00 – 60.00)**  24.00(6.00 – 48.00)  12.00(6.00 – 48.00)  12.00(6.00 – 24.00)  48.00(12.00 – 96.00)  12.00(2.00 – 36.00)  36.00 (12.00 – 60.00) | **36.00(12.00 – 60.00)**  24.00(6.00 – 48.00)  12.00(7.50 – 48.00)  12.00(6.00 – 24.00)  60.00(24.00 – 114.0)  12.00(4.00 – 36.00)  36.00 (12.00 – 72.00) | **24.00 (6.00 – 36.00)**  6.00(3.75 – 24.00)  24.00(6.00 – 30.00)  10.00(5.25 – 21.00)  24.00(12.00 – 84.00)  1.00(1.00 – 18.00)  24.00(7.50 – 54.00) | **0.0132**  0.0277  0.5733  0.3994  0.2188  0.0645  0.2355 |
| **Urinary**  Urgency  Nocturia | **36.00(12.00 – 60.00)**  36.00 (12.00 – 60.00)  36.00 (12.00 – 72.00) | **36.00(12.00 – 60.00)**  36.00 (12.00 – 60.00)  36.00 (12.00 – 72.00) | **24.00(12.00 – 60.00)**  24.00(12.00 – 45.00)  36.00(12.00 – 60.00) | **0.4259**  0.3666  0.7559 |
| **Sexual function**  Loss of libido/hypersexuality  Sexual dysfunction | **48.00(24.00 – 120.0)**  48.00(24.00 – 120.0)  48.00(24.00 – 120.0) | **48.00(24.00 – 120.0)**  48.00(21.00 – 123.0)  48.00(24.00 – 120.0) | **60.00(24.00 – 75.00)**  60.00(36.00 – 66.00)  60.00(24.00 – 84.00) | **0.8000**  0.9474  0.6481 |
| **Miscellaneous**  Taste/smell impairment  Weight fluctuations  Hyperhidrosis  Pain | **36.00(12.00 – 60.00)**  36.00(24.00 – 102.0)  36.00(12.00 – 60.00)  30.00(9.00 – 60.00)  36.00(12.00 – 54.00) | **36.00(12.00 – 60.00)**  36.00 (12.00 – 120.0)  24.00(12.00 – 60.00)  24.00(9.00 – 60.00)  36.00(12.00 – 60.00) | **36.00(24.00 – 72.00)**  60.00(24.00 – 96.00)  48.00(24.00 – 72.00)  36.00(7.50 – 69.00)  24.00(12.00 – 42.00) | **0.1095**  0.4102  0.1599  0.3970  0.5840 |
| IQR, 25%-75% interquartile range; mo, months; NMS, non-motor symptom; PD, Parkinson’s disease; REM, rapid eye movements. Differences in continuous variables between males and females were estimated using the Mann-Whitney U test. | | | | |
